# Human parvovirus B19 infection in hospitalized patients suspected of infection with pathogenic microorganism

**DOI:** 10.3389/fcimb.2022.1083839

**Published:** 2022-12-21

**Authors:** Junshuang Guo, Yating Wang, Mian Zhang, Hongxiang Zheng, Qiuling Zang, Peipei Huang, Lijun Wen, Dandan Song, Fan Yang, Ruirui Dong, Wang Miao

**Affiliations:** ^1^ Neuro-Intensive Care Unit of the First Affiliated Hospital of Zhengzhou University, Zhengzhou, Henan, China; ^2^ Department of Immunology, School of Basic Medical Science, Central South University, Changsha, Hunan, China; ^3^ General intensive care unit of Zhengzhou Seventh People’s Hospital, Zhengzhou, Henan, China

**Keywords:** human parvovirus B19, next-generation sequence, encephalitis, nerve damage, transfusion

## Abstract

**Background:**

Human parvovirus B19 (HPV B19) is a single-stranded DNA virus. The detection rate of HPV B19 in the blood of healthy blood donors using PCR technology was reported to be 6.323/100000. However, that among hospitalized patients suspected of being infected with a pathogenic microorganism is unknown.

**Methods:**

A retrospective analysis was conducted on 2,182 high-throughput NGS results for 1,484 inpatients admitted to the First Affiliated Hospital of Zhengzhou University from January 2020 to October 2021 who were suspected of being infected with a pathogenic microorganism, as well as on clinical data of some HPV B19-positive patients.

**Results:**

Human parvovirus B19 was detected in 39 samples from 33 patients. The positivity rate was 2.22% among patients and 1.78% among samples. HPV B19 was detected in 20 cerebrospinal fluid samples, 13 blood samples, 3 alveolar lavage fluid samples, 2 tissue samples, and 1 throat swab. Based on clinical symptoms and NGS results, 16 patients were diagnosed with HPV B19 infection. The number of HPV B19 sequences in these patients was greater than 6, and the patients showed common symptoms such as fever (14 cases), anemia (11 cases), and severe nervous system symptoms such as meningoencephalitis (9 cases) and Guillain–Barré syndrome with peripheral motor and sensory nerve axon damage (4 cases). All 16 patients had experienced events likely to lead to decreased immunity (11 had a history of trauma/surgery/major disease, 4 had a history of precursor infection, and 3 had used immunosuppressants) and 7 had a history of blood transfusion during hospitalization. After treatment with antiviral drugs (12 cases) and intravenous human immunoglobulin (3 cases), of the 16 patients, 14 patients improved.

**Conclusion:**

The HPV B19 infection rate in hospitalized patients suspected of microbial infection was 2.22%. Most patients with HPV B19 infection had a history of low immunity and blood transfusion. HPV B19 could be detected in various bodily fluids and tissues (especially cerebrospinal fluid) using NGS. Patients with severe HPV B19 infection may have nervous system damage such as Guillain–Barré syndrome and meningoencephalitis. Early diagnosis using NGS and treatment with antiviral drugs and immunoglobulin can improve prognosis.

## 1 Introduction

Human parvovirus B19 (HPV B19) is a single-stranded DNA virus belonging to the genus *Erythrovirus* in the family Parvoviridae. It was first identified in blood donor serum Cossart et al, 1975 (Cossart et al., 1975) and has since been associated with infectious erythema, fetal edema, chronic pure red cell aplastic anemia, transient aplastic anemia crisis, acute encephalitis, and encephalopathy. HPV B19 can be transmitted through the respiratory tract, blood transfusion, and organ transplantation, among other routes. Kooistra et al. reported that 411 (6.323/100000) blood samples from 6.5 million healthy blood donors had HPV B19 DNA levels higher than 106 IU/mL (Kooistra et al., 2011), suggesting that HPV B19 may be latent in healthy people. A study on HPV B19 detection of Chinese source plasma pools showed that some source plasma pools were contaminated by viruses. HPV B19 contamination of blood products may also be the main reason for the high positive rate of HPV B19 (Jia et al., 2015). When immunity is compromised, HPV B19 infection can result in serious clinical consequences. In the nervous system, symptoms primarily present as meningoencephalitis, and mostly in individual cases (Heegaard and Brown, 2002; Young and Brown, 2004). The detection rate of HPV B19 infection among hospitalized patients suspected of infection with a pathogenic microorganism, especially those with severe disease or undergoing major surgery, remains unknown.

The detection methods used in previous studies mainly included virus isolation and PCR-based detection of viral DNA. However, the clinical application of metagenome next-generation sequencing (mNGS) has greatly improved the detection rate of microbial infection in patients and has also enabled early diagnosis and treatment. It has been reported that one case of acute viral encephalitis caused by HPV B19 infection was detected by mNGS. Previous studies were mostly case reports, with fewer case series (Cao and Zhu, 2020).

## 2 Materials and methods

From January 2020 to October 2021, the First Affiliated Hospital of Zhengzhou University sent 2,182 samples from 1,484 hospitalized patients suspected of being infected with a pathogenic microorganism for high-throughput NGS based on the Illumina platform at Beijing Genskey Medical Technology Co., Ltd. Microbial nucleic acid sequences in the samples were compared with those of existing microorganisms in the database for identification. The clinical manifestations and laboratory indicators of the identified HPV B19-infected patients were analyzed. If the clinical manifestations were consistent with those of HPV B19 infection, a diagnosis of clinical HPV B19 infection was made. The clinical data of HPV B19-positive cases were also analyzed, The sequenced data have been deposited into the National Center for Biotechnology Information (NCBI) BioProject database with SRA accession number SRP408229.

### 2.1 Ethics statement

This study was approved by the Ethics Committee of the First Affiliated Hospital of Zhengzhou University (2021-KY-0967).

## 3 Results

A total of 2,182 samples from 1,484 hospitalized patients suspected of being infected with a pathogenic microorganism were submitted for examination. Of the 2,182 samples, 887 (40.7%) were of cerebrospinal fluid, 654 (30.0%) were of blood, 372 (17.0%) were alveolar lavage fluid samples, 77 (3.5%) were tissue samples, 50 (2.3%) were sputum samples, 28 (1.3%) were of pus, 22 (1.0%) were pleural fluid samples, 18 (0.8%) were of ascites, 8 (0.4%) were pericardial effusion samples, 5 (0.2%) were throat swabs, 4 (0.2%) were urine samples, and 57 (2.6%) were of other types. Among the 1,484 inpatients suspected of microbial infection, 33 were positive for HPV B19 infection based on NGS results, representing a positivity rate of 2.22%. Among the 2,182 samples, 39 were positive for HPV B19, denoting a positive rate of 1.78%. Of these, 20 were cerebrospinal fluid samples from 18 patients, 13 were blood samples from 11 patients, 3 were alveolar lavage fluid samples from 3 patients, 2 were tissue samples from 2 patients, and 1 was a throat swab from 1 patient.

**Figure 1:**
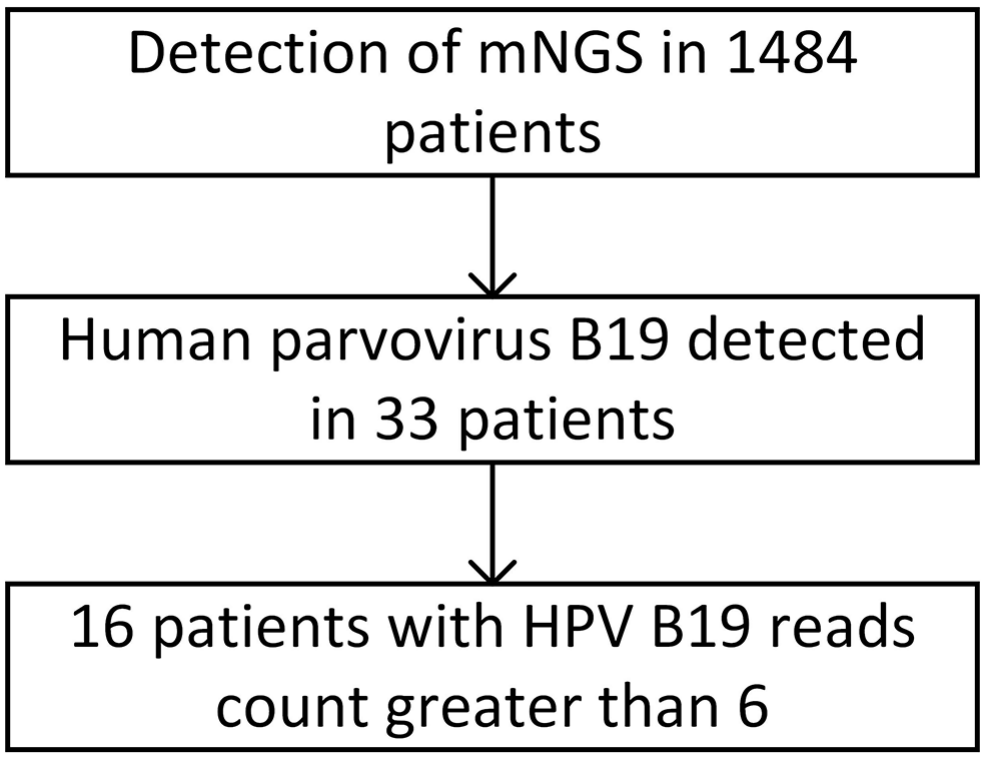
Patient screening flowchart.

The number of HPV B19 read counts in the 33 patients with HPV B19 infection ranged from 1 to 1×10^7^. Sixteen patients showed clinical symptoms of HPV B19 infection, such as fever, anemia, and nervous system involvement, to varying degrees and were diagnosed with clinical HPV B19 infection. The number of HPV B19 sequences in the 22 samples from these 16 patients (11 cerebrospinal fluid samples, 9 blood samples, 1 alveolar lavage fluid sample, and 1 throat swab) was ≥6. The number of HPV B19 sequences in the 17 samples from the other 17 patients (9 cerebrospinal fluid samples, 4 blood samples, 2 alveolar lavage fluid samples, and 2 tissue samples) was ≤3 and the clinical symptoms were not, or could not be determined to be, associated with parvovirus infection (**Figure 1**).

Of the 16 patients diagnosed with HPV B19 clinical infection, 10 were male and 6 were female. All 16 patients had experienced events leading to decreased immunity (4 cases of pre-infection, 11 cases of early disease, 6 cases of surgery, 3 cases of immunosuppressant use, and 1 delivery) while 7 had a history of blood transfusion during hospitalization. The clinical manifestations related to HPV B19 infection included 13 cases of fever, 11 cases of anemia (HPV B19 viremia), 9 cases of meningoencephalitis, and 4 cases of peripheral nerve damage (Guillain–Barré syndrome [GBS]). Case 1 had meningoencephalitis with GBS and HPV B19 viremia. Among the 16 patients, 12 were admitted to the neurological ICU and showed neurological signs and symptoms (**Table 1**).

**Table 1:** Clinical data of patients infected with human parvovirus B19.

Patient No./Sex/Age(years)	Prodromal symptom	Prophase disease	History of surgery	Neuro ICU patient	Immunosuppressant	History of blood transfusion	Fever	Anemia	Clinical manifestation	Sample and reads count	HPV B19 infection-related manifestations	Treatment plan	Score at the most serious condition	Score at discharge
1/M/60	Cold	Mitral valve prolapse	Heart valve replacement	Yes	No	Yes	Yes	Yes	Paralysis of limbs and respiratory muscles, disturbance of consciousness, signs of meningeal irritation	Blood*: 13,953,069; Cerebrospinal fluid*: 832	GBS, viral meningoencephalitis, viremia	Ganciclovir, glucocorticoid, IVIG	Hughes: 5	Hughes: 5 (Hughes score at 6 months after discharge: 2)
2/M/12	Cold	No	No	Yes	No	Yes	Yes	Yes	Quadriplegia	Blood: 19008779	GBS, viremia	Ganciclovir	Hughes: 5	Hughes: 5
3/F29	No	Childbirth	No	Yes	No	No	No	No	Paralysis of limbs and respiratory muscles	Cerebrospinal fluid: 9	GBS	Penciclovir, IVIG	Hughes: 5	Hughes: 3
4/M/40	Cold	No	No	Yes	No	No	No	No	Paralysis of limbs and respiratory muscles	Cerebrospinal fluid: 6	GBS	IVIG, ganciclovir, immunoadsorption	Hughes: 5	Hughes: 4 (Hughes score at 6 months after discharge: 0)
5/M/14	No	Fracture	Fracture reduction and fixation	Yes	No	Yes	Yes	Yes	Disorder of consciousness, signs of meningeal irritation	Blood: 209513	Viral encephalitis, viremia	Ganciclovir	GCS: 6	GCS: 13
6/M/30	No	Cerebral hemorrhage	Removal of intracranial hematoma	Yes	No	No	Yes	Yes	Disorder of consciousness, signs of meningeal irritation	Cerebrospinal fluid: 3866; Blood: 36	Viral encephalitis, viremia	Ganciclovir	GCS: 6	GCS: 10
7/F/48	No	Cirrhosis	Allogeneic liver transplantation	No	Yes	Yes	Yes	Yes	/	Blood: 139	Viremia	Immunosuppressant	mRS: 5	mRS: 1
8/F/36	No	Sjogren’s syndrome	Lymph node dissection	No	Yes	No	Yes	Yes	/	Pharyngeal swab: 6	Respiratory infection, viremia	Immunosuppressant	mRS: 3	mRS: 1
9/F/74	No	Interstitial lung disease, anti-synthetase syndrome	No	No	Yes	No	Yes	No	/	Alveolar lavage fluid: 25	Pulmonary infection	Immunosuppressant	mRS: 5	mRS: 1
10/F/37	Tuberculosis infection	Tuberculous meningoencephalitis	No	Yes	No	Yes	Yes	Yes	Disorder of consciousness, signs of meningeal irritation	Blood: 6	Viral encephalitis, viremia	Anti-infection treatment	GCS: 11	GCS: 15
11/M/68	No	No	No	No	No	No	No	No	Disorder of consciousness, signs of meningeal irritation	Cerebrospinal fluid: 129	Viral encephalitis	Ganciclovir	GCS: 15	GCS: 15
12/M/50	No	Cerebral hemorrhage	Removal of intracranial hematoma	Yes	No	Yes	Yes	Yes	Disorder of consciousness, signs of meningeal irritation	Cerebrospinal fluid*: 474478	Viral encephalitis, viremia	Ganciclovir	GCS: 6	GCS: 9
13/M/66	No	Craniocerebral injury	No	Yes	No	No	Yes	Yes	Disorder of consciousness, signs of meningeal irritation	Cerebrospinal fluid: 433	Viral encephalitis, viremia	Ganciclovir	GCS: 6	GCS: 13
14/F/57	No	No	No	Yes	No	No	Yes	No	Disorder of consciousness, signs of meningeal irritation	Cerebrospinal fluid: 15	Viral encephalitis	Ganciclovir	GCS: 8	GCS: 15
15/M/37	No	Multiple organ failure	No	Yes	No	Yes	Yes	Yes	/	Blood: 368	Viremia	Ganciclovir	mRS: 5	mRS: 1
16/M/63	No	Cerebral hemorrhage	No	Yes	No	No	Yes	Yes	Disorder of consciousness, signs of meningeal irritation	Cerebrospinal fluid: 10	Viral encephalitis, viremia	Ganciclovir	GCS: 3	GCS: 5

F, female; M, male; IVIG, intravenous immunoglobulin; GBS, Guillain-Barre syndrome; GCS, Glasgow Coma Scale; mRS, modified Rankin score; *, The patient received multiple next generation sequencing of cerebrospinal fluid or blood. Patient 1 received three blood NGS tests, the results were 13953069, 9006108, 955 respectively, and two cerebrospinal fluid NGS tests, the results were 832 and 488 respectively. Patient 12 received two tests of cerebrospinal fluid NGS, and the results were 474478 and 5636649 respectively.

Twelve patients were treated with ganciclovir or penciclovir and 3 were treated with human immunoglobulin. Fourteen patients were discharged with improvement.

The six patients positive for HPV B19 in blood all had a history of blood transfusion. Five of these patients had more than 100 viral sequences in their blood and their symptoms appeared after blood transfusion. Among the nine patients positive for HPV B19 in cerebrospinal fluid, only two had a history of blood transfusion. The number of HPV B19 sequences in the cerebrospinal fluid of case 6, who had no history of blood transfusion, was significantly higher than that in blood, while the number of HPV B19 sequences in the blood of case 1, who had a history of blood transfusion, was significantly higher than that in cerebrospinal fluid. Both patients had a fever and decreased hemoglobin. (See supplementary materials for some medical records)

## 4 Discussion

Our results showed that, at approximately 2.22%, the HPV B19 infection rate in hospitalized patients suspected of microorganismal infection as detected by NGS was higher than that previously reported for healthy blood donors based on PCR technology. Hospitalized patients with low immunity were at greater risk of HPV B19 infection. Besides fever and anemia, nervous system involvement was also a primary clinical manifestation of HPV B19 infection. This may have been due to HPV B19 entering the brain *via* the blood–brain barrier or latent HPV B19 reactivation in the nervous system. For patients with a very high number of viral sequences, the possibility of infection *via* blood transfusion could not be excluded. Through the application of antivirals (ganciclovir) and/or immunoglobulin therapy, most patients achieved a good prognosis.

### 4.1 The detection rate of HPV B19 infection can be improved by screening samples of hospitalized patients with suspected infection using NGS

We found that the detection rate of HPV B19 infection by NGS in hospitalized patients suspected of microorganismal infection was 2.22%, which is higher than that reported for PCR-based detection of HPV B19 in serum (0.6%–0.003%) (Heegaard and Brown, 2002). Several reasons may explain this difference. Testing for HPV B19 DNA has traditionally been performed on volunteer blood donors, whereas our subjects were hospitalized patients suspected of infection. This suggests that the infection rate of HPV B19 is higher in hospitalized patients suspected of infection than in healthy people, and vigilance against HPV B19 infection in such patients must be strengthened. In this study, the patients with HPV B19 infection all had experienced events leading to decreased immunity, such as more serious early disease, history of surgery, history of precursor infection, and history of immunosuppressant use. These observations suggest that low immunity is the main factor underlying susceptibility to HPV B19 infection. NGS has high detection accuracy and sensitivity, and can thus improve the detection rate of viruses, help with diagnostic and treatment decisions, and can also aid in the evaluation of treatment effects through the dynamic monitoring of the viral load of patients. Although NGS can improve the efficiency and accuracy of diagnosis, its high cost and technical requirements limit its possibility as a census item. For practical purposes, the samples tested in this study included cerebrospinal fluid, blood, tissue, and respiratory secretions. The samples were derived from a wide range of sources, which likely improved the detection rate among hospitalized patients suspected of infection.

### 4.2 Possible pathways by which HPV B19 enters the nervous system

In this study, NGS was used for the first time to quantify the number of HPV B19 sequences in the cerebrospinal fluid of patients. The number of viral sequences in the cerebrospinal fluid of some patients was very high, suggesting that HPV B19 may directly enter the central nervous system. Of the 16 patients diagnosed with HPV B19 clinical infection in this study, 12 had nervous system involvement. This indicated that the nervous system was vulnerable to infection by HPV B19, which enriches the infection range of parvoviruses. Previous study showed that nervous system symptoms caused by HPV B19 infection were meningoencephalitis (Watanabe and Kawashima, 2015) and Guillain–Barré syndrome (Minohara et al., 1998), and most cases are confirmed by the detection of viral DNA or antibody in blood, in a few cases, HPV B19 DNA is also detected in cerebrospinal fluid (Watanabe and Kawashima, 2015). Given its high detection sensitivity, NGS technology has improved the HPV B19 detection rate in cerebrospinal fluid, and is helpful for the diagnosis of patients with central nervous system HPV B19 infection.

**Figure 2:**
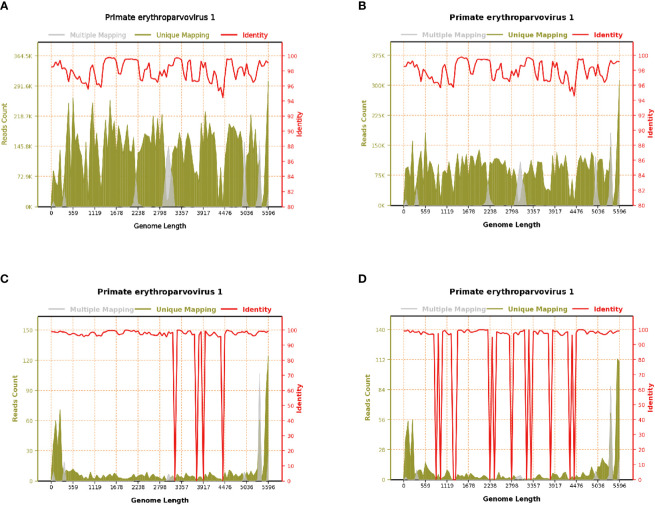
Parvovirus B19 genome coverage of case 1. **(A)** Blood on August 19, 2020; reads count: 13,953,069, genome coverage: 100% (5596/5596). **(B)** Blood on August 22, 2020; reads count: 9,006,108, genome coverage: 100% (5596/5596). **(C)** Blood on August 31, 2020; reads count: 955, genome coverage: 98.427448% (5508/5596). **(D)** Cerebrospinal fluid on August 28, 2020; reads count: 832, genome coverage: 91.976412% (5147/5596).

There are two possible mechanisms *via* which HPV B19 can affect the central nervous system. The first involves directly transversing the blood–brain barrier. In this study, 12 patients had nervous system symptoms, and a greater number of HPV B19 sequences was detected in the cerebrospinal fluid than in the blood of these patients, with viral sequence coverage of some patients exceeding 90% (**Figure 2**). The number of HPV B19 sequences in the blood of case 1 was higher than that in cerebrospinal fluid, suggesting that the virus in the blood may enter the central nervous system through the blood–brain barrier. The second involves the activation of latent virus. However, there is a lack of direct results of animal or cell experiments. It is expected that more researchers will carry out experimental verification for the above conjecture. The 12 patients presenting with meningoencephalitis or GBS in this study all had severe pre-existing diseases, a history of surgery, prodromal infection, a history of immunosuppressant use, or any other condition that lowered their immune functions. Some patients (cases 3, 4, 11, and 14) did not have anemia but had evident neurological symptoms. HPV B19 has been reported in blood, bone marrow, fetal liver, and other tissues and organs of healthy blood donors (Heegaard and Brown, 2002). Accordingly, it cannot be excluded that HPV B19 could also colonize the central nervous system during infection, and the associated symptoms may be caused by the activation and proliferation of latent HPV B19 under conditions of diminished immunity.

### 4.3 The possible mechanism of HPV B19 infection in hospitalized patients suspected of infection

HPV B19 shows a marked tropism for erythroid progenitor cells by combining with cell receptor P antigen (Brown et al., 1993), Eleven of the 16 patients diagnosed with HPV B19 clinical infection in this study developed anemia, and the proportion of anemia due to HPV B19-mediated erythrocyte damage was high. This may be related to the fact that the patients in this study experienced events that led to a decline in immunity and could not produce protective antibodies in time. Under these conditions, the virus continued to replicate and proliferate, resulting not only in anemia but also in damage to the nervous system. Eight patients in this study had more than 100 viral sequences, 6 had viral encephalitis, and 2 had GBS. The number of viral sequences in cerebrospinal fluid was also high. These findings suggest that the virus can directly damage the nervous system. One study reported that HPV B19 can directly invade the central nervous system and that the viral load in cerebrospinal fluid is related to the clinical symptoms of encephalitis in patients (Oshima et al., 2008). In this study, the levels of the cytokines IL-6 and IL-8 in the cerebrospinal fluid of cases 1, 3, and 10 were significantly increased than normal(**Table 2**), suggesting that inflammatory cytokines may also play an important role in HPV B19-mediated damage to the nervous system (Zang et al., 2021; Amorim et al., 2022). Humoral immunity may also be involved in the damage to the nervous system. Four patients in this study had GBS, and electromyography suggested that the damage to the peripheral nerves involved not only the myelin sheath but also more extensively the axons. The rate of cerebrospinal fluid synthesis in the sheath was increased in three patients. Studies have shown that the pathogenesis of GBS is mostly related to antibody-mediated humoral immune damage (Willison et al., 2016). In brief, the mechanism underlying how HPV B19 damages the nervous system may be due to direct effects, cytokine storm, or the production of pathogenic antibodies (Barah et al., 2003; Douvoyiannis et al., 2009; Barah et al., 2014).

**Table 2:** Results of the levels of lymphocyte subsets and cytokines in patient 1.

Specimen	LYM#	CD3+T#	CD3+CD4+T#	CD3+CD8+T#	CD19+#	NK#	Specimen	IL-6	IL-8
Blood	1704.0	1264.0	778.0	391.0	333.0	64.0	Blood	286.6	0.9
Blood	813.0	419.3	337.2	70.4	381.8	15.5	Blood	305.9	280.1
Blood	478.2	258.3	187.6	62.6	205.7	10.0	CSF	78.1	1396.8
CSF	12.8	10.2	5.6	4.6	1.0	0.5	CSF	370.8	732.4
CSF	8.7	4.7	2.3	2.3	2.3	0.3	Blood	300.0	248.2

LYM# (/UL): absolute number of lymphocytes; CD3+T# (/UL): absolute number of total T lymphocytes; CD4+T# (/UL): absolute number of auxiliary/induced T lymphocytes; CD8+T# (/UL): absolute number of inhibitory/cytotoxic T lymphocytes; CD19+# (/UL): absolute number of B lymphocytes; NK# (/UL): the absolute number of NK lymphocytes; IL: interleukin; CSF: cerebrospinal fluid. The unit of IL-6 and IL-8 is pg/mL.

### 4.4 The treatment of HPV B19 clinical infection in hospitalized patients suspected of infection

There is as yet no consensus regarding the treatment of HPV B19 clinical infection. However, studies have shown that intravenous injection of human immunoglobulin or glucocorticoid can achieve partial effects (Watanabe and Kawashima, 2015). In this study, 12 patients were treated with ganciclovir or penciclovir, and 10 achieved satisfactory results. Those that did not (cases 1 and 2) had a serious disease in the early stages of infection, which masked the symptoms of viral infection. Diagnosis was delayed and antiviral treatment was started relatively late, resulting in a high viral load. As nucleotide triphosphate analogs, ganciclovir can competitively bind with viral DNA polymerase and thereby preventing the replication of the virus (Jockusch et al., 2020). Ganciclovir and IVIG have a good effect in treating encephalitis and GBS caused by other virus (Miao et al., 2021; Zang et al., 2021). In this study, 2 patients were administered ganciclovir/penciclovir combined with human immunoglobulin for the treatment of GBS caused by HPV B19 infection, with good results, suggesting that the ganciclovir/human immunoglobulin combination has the potential for use in the treatment of patients with HPV B19 clinical infection.

### 5 Conclusion

The infection rate of HPV B19 among hospitalized patients suspected of microorganismal infection was found to be 2.22%. Most patients with HPV B19 infection had a history of low immunity and blood transfusion. HPV B19 can be detected in various bodily fluids and tissues using NGS. Patients with severe HPV B19 infection may present with nervous system involvement, such as GBS and meningoencephalitis. Early diagnosis *via* NGS and treatment with antiviral drugs and/or immunoglobulin can improve prognosis. The incidence rate of parvovirus infection is low, and there are few clinical cases. This study is a retrospective analysis of small samples, lacking control, and only statistical description has been carried out. More researchers need to cooperate for further research.

## Data availability statement

The datasets presented in this study can be found in online repositories. The names of the repository/repositories and accession number(s) can be found below: NCBI, SRA: SRP408229.

## Ethics statement

The studies involving human participants were reviewed and approved by This study was approved by the Ethics Committee of the First Affiliated Hospital of Zhengzhou University (2021-KY-0967). Written informed consent to participate in this study was provided by the participants’ legal guardian/next of kin.

## Author contributions

WM conceived the study and supervised this work. JG and YW conceived the study, organized and statistical data, and drafted the manuscript. MZ, HZ, QZ, PH, LW, DS, FY, and RD conceived the study and assisted in collecting data. All authors reviewed and approved the final manuscript. All authors contributed to the article and approved the submitted version.
